# Toward Rapid Aspartic Acid Isomer Localization in Therapeutic Peptides Using Cyclic Ion Mobility Mass Spectrometry

**DOI:** 10.1021/jasms.2c00053

**Published:** 2022-05-24

**Authors:** Katherine Gibson, Dale A. Cooper-Shepherd, Edward Pallister, Sophie E. Inman, Sophie E. Jackson, Viv Lindo

**Affiliations:** † Yusuf Hamied Department of Chemistry, University of Cambridge, Cambridge CB2 1EW, U.K.; ‡ Analytical Sciences, BioPharmaceuticals Development, R&D, AstraZeneca, Cambridge CB21 6GH, U.K.; § Waters Corporation, Wilmslow SK9 4AX, U.K.

## Abstract

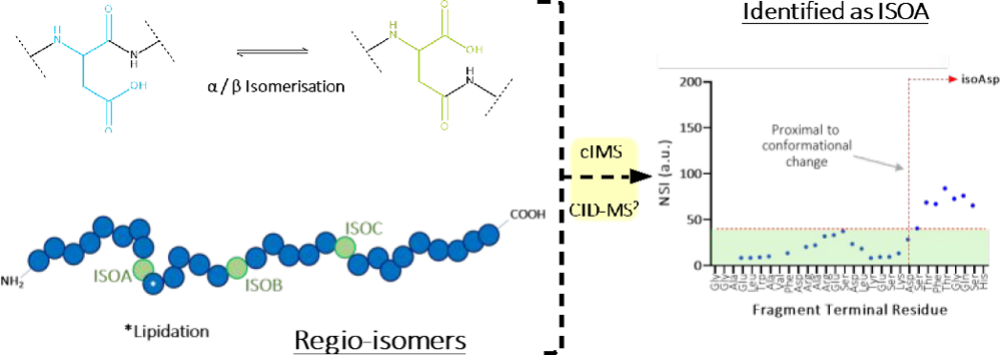

There is an increasing emphasis on the critical evaluation of interbatch purity and physical stability of therapeutic peptides. This is due to concerns over the impact that product- and process-related impurities may have on safety and efficacy of this class of drug. Aspartic acid isomerization to isoaspartic acid is a common isobaric impurity that can be very difficult to identify without first synthesizing isoAsp peptide standards for comparison by chromatography. As such, analytical tools that can determine if an Asp residue has isomerized, as well as the site of isomerization within the peptide sequence, are highly sought after. Ion mobility-mass spectrometry is a conformation-selective method that has developed rapidly in recent years particularly with the commercialization of traveling wave ion mobility instruments. This study employed a cyclic ion mobility (cIMS) mass spectrometry system to investigate the conformational characteristics of a therapeutic peptide and three synthetic isomeric forms, each with a single Asp residue isomerized to isoAsp. cIMS was able to not only show distinct conformational differences between each peptide but crucially, in conjunction with a simple workflow for comparing ion mobility data, it correctly located which Asp residue in each peptide had isomerized to isoAsp. This work highlights the value of cIMS as a potential screening tool in the analysis of therapeutic peptides prone to the formation of isoAsp impurities.

## Introduction

There exists a vast array of therapeutics centered around biologics, encompassing antibodies, antibody–drug conjugates, and mRNA to name but a few.^1−3^ Not least of all are peptides; which are relevant to a number of therapeutic areas and drug discovery programs,^4,5^ e.g., glucagon-like peptide-1 receptor agonists used in the treatment of type 2 diabetes.^6^ Noncovalent interactions and structural complementarity are critical to biological systems information transfer and intermolecular recognition. It is therefore unsurprising that peptides, with their innate flexibility and access to numerous conformational ensembles, are attractive moieties for drug discovery.

A significant difference with small molecule drugs is that peptides are more prone to physical instability having, in some cases, a high propensity for self-assembly and aggregation.^7^ Aggregation poses a major concern in the development of peptide and protein pharmaceuticals due to the complexity of potential adverse immune responses from aggregates^8,9^ and, of course, any uncertainty over the efficacy or promiscuity of the aggregated species. In particular, the presence of impurities or seeds (preformed short fibrils) can trigger and, in some cases, modify fibrillation with respect to both the rate and the type/structure of fibrils formed.^10−12^

**Figure 1:**
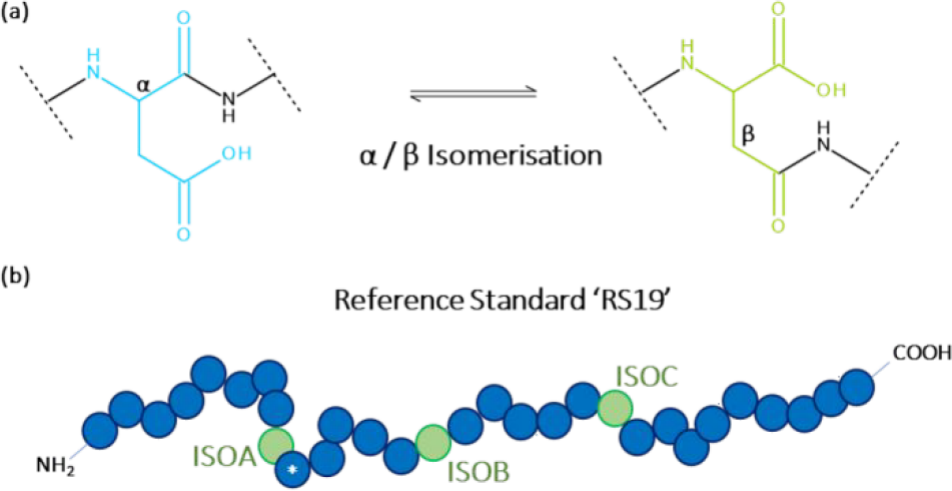
Isomerization of aspartic acid and location of aspartic acids in RS19. (a) Isomerization of α-aspartic acid residue that results in the formation of a β-aspartic acid. (b) Representation of RS19 (World Patent WO2017153575A1, 2017) with the three potential isomerization sites (Asp residues) highlighted in green at positions 9 (ISOA), 15 (ISOB), and 21 (ISOC). The site of lipidation is also shown (*).

Amino acid isomerization can alter a protein or peptide’s physical stability, conformation, or activity.^13−15^ Isomerization can occur as a byproduct of peptide synthesis^16^ or spontaneously; in particular, the formation of isoAsp from either Asp or deamidated Asn via a common succinimide intermediate^17^ is well-known and introduces an additional methylene into the peptide backbone (Figure 1). Isomerized peptides have been shown in many settings to change the properties of a peptide^18−21^ and therefore must be characterized fully as potential impurities before clinical development. Therefore, the development of techniques that can identify the location of isoAsp isomers and differentiate isomers from their Asp counterparts are crucial.

Reversed-phase liquid chromatography coupled to mass spectrometry (LC–MS) is an obvious option to determine the presence of impurities, their molecular formula, and relative hydrophobicity. Fragmentation of peptides, with MS^2^, can routinely detect various isomers; however, impurities in which there has been an isomerization of an Asp to isoAsp are challenging to detect. MS^2^ with traditional collision-induced dissociation (CID) yields fragment ions that remain isobaric and are therefore ambiguous. Sophisticated and more niche fragmentation methods have been successful at differentiating isomers including isoAsp and Asp from each other and deamidation products.^22−24^ Electron transfer dissociation (ETD), for example, has been conducted on Asp- and isoAsp-containing peptides^25^ but traditionally uses hazardous reagents, produces low intensity fragments, and is known to suffer from poor fragmentation efficiency from analytes with lower charge densities.^26^ This and many other techniques lack the appropriate sensitivity, speed, and resolution required for batch-to-batch quality control. Pairing MS with a structure-based differentiation technique, like ion mobility-MS (IMS),^27^ is a natural choice for identifying isomers with the same chemical formula, hence, *m*/*z*, but potentially different molecular conformations. IMS has already demonstrated capability in helping distinguish trace impurities in pharmaceuticals.^28^

Ion mobility experiments involve separating ions in the gas phase based primarily on both their charge and their rotationally averaged collision cross sectional area (CCS, Ω). The technique is often used in conjunction with time-of-flight mass spectrometry (typically QTOF) to provide information on both mass and structure. For details of IMS, its application, and some of the experimental methods available, interested readers are directed to reviews available elsewhere.^29,30^

In traveling wave ion mobility (TWIMS) experiments such as those using the Synapt G2 (Waters Corp.) commercial instrument, the ions pass through a defined length of a stacked ring ion guide (SRIG) composed of circular electrodes confining ions radially by an applied radio frequency voltage. Ions are pushed through, against the buffer gas, by a direct current (DC) pulse which travels along the series of contiguous electrodes like a wave,^31,32^ the principle being that larger, lower charged ions take longer to travel through the mobility device. This is because larger ions “tumble over” DC waves that are sufficient to push smaller ions forward. Hence, conformations that result in an expanded ion rather than a compact one exit the drift cell and arrive at the mass detector later. Mobilities can be represented by arrival time distributions (ATD), with the width, asymmetry and peak maxima of these ATD providing information on the relative conformation of the ions. Wider distributions typically represent one of two things, either multiple ions that have nonresolvable drift times and hence are likely to be structurally very similar or conformers of one species that are interconverting on the time scale of the mobility separation.

A new development in IMS is the commercial release of the SELECT SERIES Cyclic IMS instrument from Waters Corp. The flagship feature of cyclic IMS (cIMS) is the potential for passing ions around the circular “racetrack” of the cyclic T-wave device an infinite number of times. Improved resolution of the cyclic system compared to a linear traveling wave device, which is limited by the length of the mobility cell, has already been highlighted a number of times despite being a relatively new technology.^33,34^ The cyclic system is limited only when ions become so well resolved that the fastest overtakes the slowest, somewhat analogous to cyclists being lapped in a velodrome. The technique and instrumental details are described in depth by Giles et al.,^35^ but notably, the SELECT SERIES Cyclic IMS allows ions separated on account of their mobility (fragments or whole ions) to be selected and reinjected into the cyclic array to tease out the finer features of their conformational differences. This technique (termed IMS^n^)^35^ is not possible with the earlier linear versions of the TWIMS instruments.

In this work, we present the use of cIMS for differentiating a model system of three synthetic isomers (ISOA, ISOB, and ISOC which are isoAsp9, isoAsp15, and isoAp21, respectively) of a therapeutic peptide from their nonisomerized reference standard, RS19.^36^ Of the three isomeric peptides, each contained a single aspartic acid residue as an isoAsp, and the sample set was chosen with two main objectives in mind. First, the ATD of each isomer after multiple passes was used as a comparator to differentiate the isomers from RS19, thereby evaluating cIMS as an analytical technique sensitive enough to differentiate potential impurities that are chemically similar. Second, the resolution of cIMS meant that subtle differences in the ATDs of fragment ions was capable of identifying the position of the β-aspartic acid. Thus, pseudosequencing of the isomeric modification was achieved by a pairwise comparison of fragment ion ATDs which accounted for differences not only in arrival time in a manner similar to that outlined by Jia et al.^37^ and Tomczyk et al.^38^ but also peak shape. This new processing method, called (normalized subtraction integration, NSI), was developed to provide a quantitative measure to describe how different two ATDs were from each other and was applied to RS19 and its three singly isomerized isoAsp counterparts. The workflow proposed here is a step toward a more objective analysis of high-resolution ion mobility data of biomolecules, with the potential to incorporate it into a more automated workflow through combination with chromatographic separation to give LC-CID-cIMS.

## Experimental Section

### Sample Preparation

All peptides were supplied as lyophilized powders by AstraZeneca. Peptides were solubilized in 20 mM ammonium acetate (pH 6.4) prior to filtration through a 0.22 μm syringe filter (Millex-GV) and dilution to the required concentration (5–9 μM). Solutions were aliquoted and frozen at −80 °C for storage. Samples were thawed only once prior to infusion by nESI or injection to LC. Peptide isomer identities were known prior to data analysis. ISOA was isomerized at Asp9, ISOB was isomerized at Asp15, and ISOC was isomerized at Asp21.

### Instrumentation

Ion mobility mass spectra were acquired using a SELECT SERIES Cyclic IMS (Waters) instrument using PicoTip Glass Tip coated capillaries (NewObjective). Native spray conditions were as follows: capillary 1–1.2 kV, cone 40 V, source temperature 100 °C. All other instrument parameters were used as default. The cyclic ion mobility cell was operated at a pressure of 1.8 mbar nitrogen, with a static traveling wave height of 15 V. Single pass cIMS experiments were performed with an inject time of 10 ms and separate time of 2 ms. The number of passes was increased up to four by increasing the separate time accordingly.

Liquid chromatographic separation of the synthetic peptides was performed on an ACQUITY I-Class UPLC (Waters) equipped with an ACQUITY Premier 1.7 μm, 2.1 × 100 mm CSH C18 column operated at 60 °C. Mobile phases were water (A) and acetonitrile (B) each with formic acid added to a final concentration of 0.1%. The proportion of mobile phase B was increased from 35–45% over 8 min for separation of the isomeric peptides.

For the infusion-based CID-cIMS experiments, the [M + 3H]^3+^ ion (1242 *m*/*z*) of each peptide was selected in the resolving quadrupole before collisional activation at 75 or 80 V in the trap collision cell. The resulting product ions were subjected to a single pass only for this analysis. For the LC-CID-cIMS experiments, fragment ions were generated after quadrupole isolation of the [M + 4H]^4+^ ion (933 *m*/*z*) using a trap collision energy of 45 V in single pass cIMS mode. A combination of +1, + 2 and +3 fragments were analyzed for each data set after a single pass of the cyclic device, but each isomer was always compared to the corresponding *m*/*z* of RS19. On occasion, for less abundant fragments, more than just the monoisotopic peak had to be extracted to generate an acceptable ATD.

### Software

Mass spectra were processed using Masslynx v4.2 (SCN 1016 and SCN1007) and DriftScope v3 and v2.8. Arrival time distributions were replotted in either Microsoft Excel (Office 365) or GraphPad Prism (v. 9.0.0) after extracting chromatogram lists and plotting intensity as a percentage of the maximum value, per mobilogram, in the interval range extracted. Calculations of the fragment ion ATD similarity (NSI) were conducted using a Python script prepared in house using Python (v.3.9) and the HTML-based Jupyter Notebook. (v. 6.4.0) The script was written using pandas^39,40^ (v. 1.3.1) and tkinter (v. 8.6) to import mobility data as csv files which had previously been exported as chromatograms from Masslynx where the *x* axis was arrival time (in milliseconds) and the *y* axis intensity (*I*_*m***/***z*_) For each ion *I*_*m***/***z*_, the extracted intensity was normalized to a percentage intensity (relative intensity, RI) at time *t* by dividing by the maximum extracted intensity (*I*_max**,***m***/***z*_) for that specific ion (eq 1) across all time points. It was this relative intensity from the isomer ion that was subtracted from the reference ion relative intensity at each time point to give the difference, *D*. As this number could be positive or negative, dependent on how the two ion ATDs differed, the absolute value was calculated at each time point, and finally, this was used to construct a graph of absolute relative intensity (%) versus time (ms). This plot was integrated using the trapezoid function from the scipy.integrate library to give a quantitative value (which we call the NSI) which represents the difference between that isomer ion ATD and the corresponding RS19 fragment ion ATD (eq 2). To reduce fluctuations in these values when plotted per terminal amino acid along the sequence of the peptide, a running average was plotted instead. This means that for a fragment ion ending in residue n, the NSI plotted at that n value was the average NSI for fragment *n* – 1, *n*, and *n* + 1. The workflow, from which NSI values were calculated, can be seen in Figure S1.
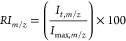
1
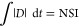
2

## Results and Discussion

### Differentiation of Precursor Ions using cIMS

RS19 and the isomers were individually solubilized in 20 mM ammonium acetate (pH 6.4) to concentrations between 5 and 9 μM before direct infusion by nESI into the SELECT SERIES Cyclic IMS. The monomeric [M + 3H]^3+^ species (the most abundant ion observed for each isomer Figure S2) was quadrupole selected and investigated, first after a single pass and then multiple passes.

**Figure 2:**
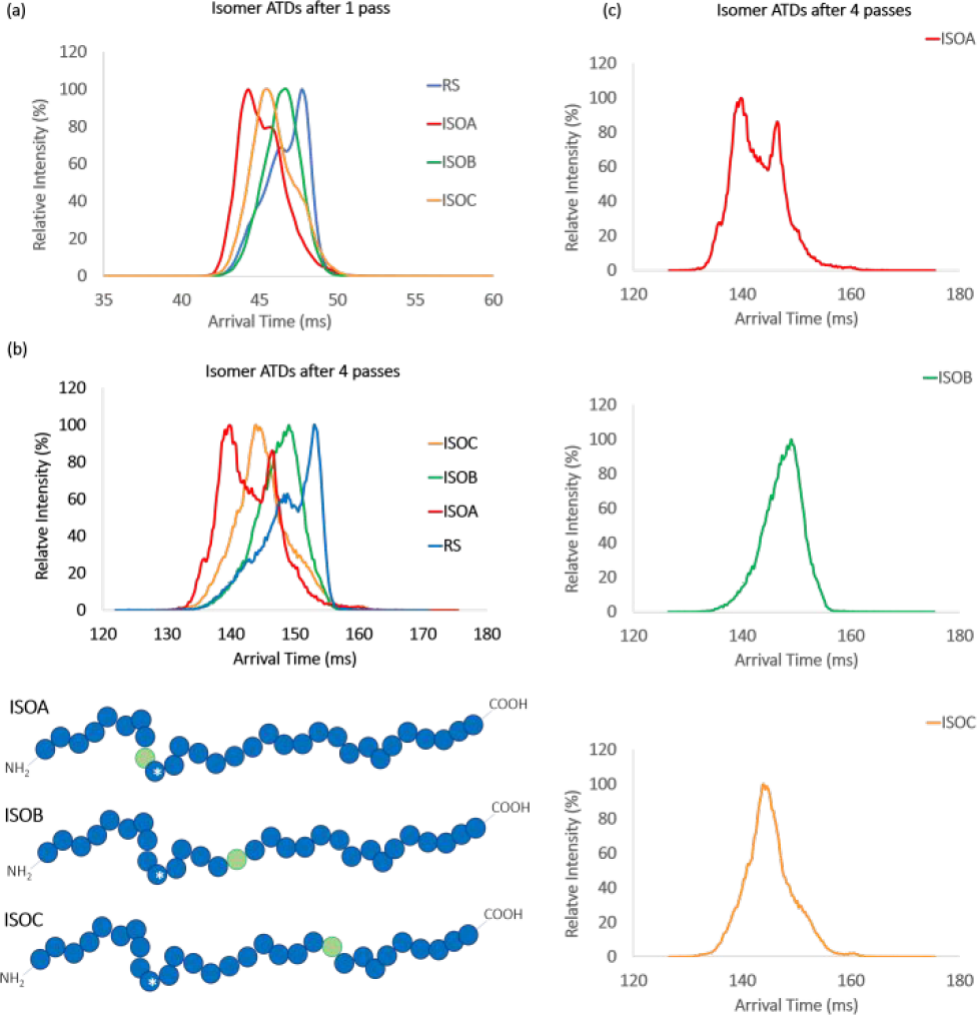
Comparison of the ATDs of individual intact isomers and the reference standard (RS19) after a single pass and four passes around the cyclic T-wave device. Subtle features in the ATDs after a single pass (a) are exaggerated after four passes (b) where it is possible to clearly differentiate one isomer from another. (c) For ISOA two distinct conformer populations were observed, but for ISOB and ISOC only one was observed. Each isomer was observed to have a different arrival time for its major conformation. The bottom left panel shows the position of the isomerized Asp residue (green) in each isomer.

There are three Asp residues in the sequence with each peptide having a single isomerization to isoAsp, except RS19, which was known to be completely in the Asp form. Even after one pass (Figure 2a), the isomers were more distinct from each other than they appeared using a linear TWIMS instrument (Figure S3). The arrival time distributions of the major peak maxima ranged from 44.28 to 47.72 ms, and shoulders were apparent in the ATD for ISOA, ISOC, and RS19. The major population for each isomer did not have the same arrival time, indicating a different conformational preference for each.

Figure 2 shows the difference between one and four cIMS passes for the [M + 3H]^3+^ ion (*m*/*z* 1242.9) with replicate data shown in Figure S4. The four pass data revealed distinct and unique ATDs in the range 130–160 ms for the [M + 3H]^3+^ monomer ions of each of the isobaric peptides (ISOA, ISOB, and ISOC). Here, the greater resolving power of four passes revealed differentiating features between isomers that were not as distinct after only a single pass (Figure S5). The most noticeable difference was that the shoulders on both ISOA and RS19 were now two distinct peaks. This is in contrast to the shoulder observed with ISOC which was not as apparent as it had been after one pass. In this case, either the second conformer population of ISOC was structurally more similar to its major population for ISOA and RS19 or the conformational populations in ISOC may have been interconverting between the two populations faster than the mobility measurement. This suggests that the populations of ISOA and RS19 were not exchanging as fast, if at all. Significantly more work is required to probe the exact energy landscape and dynamics of the multiple populations observed for each isomer, but this result exemplifies the potential of the instrument to differentiate the conformational ensembles of closely related peptide therapeutics, even after a single pass.

The shorter arrival time of the ISOA major population after four passes (139.85 ms) compared to that of the other peptides, in particular RS19 (153.01 ms), indicated a significant proportion of molecules were present in a more compact conformation. It became strikingly clear after four passes how different the conformational ensembles of ISOB and ISOC were from their regiomeric ISOA. ISOB had an arrival time most similar to RS19 and ISOC had a conformational preference for a more compact conformation (ATD 144.03 ms) compared to ISOB (149.08 ms).

cIMS provided strong experimental evidence that the site of isomerization can noticeably impact the conformational ensemble of a therapeutic peptide. Four passes around the cyclic device demonstrated that the three different isomers of RS19 are not conformationally equivalent, resulting in dissimilar ATDs. The change in conformation was not entirely unexpected as isomerization to a β-peptide introduces an additional backbone methylene which could disrupt intramolecular hydrogen bonding and could conceivably alter the preferred torsion angles of the backbone. Computational analyses of the conformational/energy landscape for the isomers have not been conducted, preventing definitive conclusions on their possible conformations, and the authors note that further investigation is required to explain *why* the conformations appear as they do. Here, we have shown how cIMS is capable of differentiating a therapeutic peptide from its potential impurities after only minutes of data acquisition.

### Site of Isomerization Located Using CID-cIMS

Fragmentation techniques, such as CID, cannot differentiate between fragments containing α- or β-Asp, as all isomers result in fragment pairs (e.g., *b*/*y* ions) with the same *m*/*z* to the nonisomerized peptide. Currently, potential impurities are synthesized as “standards” and chromatographic techniques used to compare a drug product with an impurity. This is time-consuming and resource intensive; thus, we combined CID with cIMS, allowing fragment ions to be differentiated on the basis of their mobilities (shape) despite having the same *m*/*z* ratio. As with previous studies on shorter peptides,^37,38^ only fragments containing the isomer should be different from the nonisomerized reference. It was expected that the conformational change induced by an Asp isomerization in fragment ions should be relatively more significant than in longer precursor ions. However, unlike previous studies, this analysis investigated peptides that had 30 residues, contained a conformationally flexible lipid, and, crucially also accounted for differences in ATD asymmetry as well as absolute arrival times. Accounting for ATD asymmetry may aid with interlaboratory comparisons given that arrival times can be liable to variation between instruments. Additionally, isomerization of larger molecules may induce only subtle changes, resulting in ATDs with multiple, less intense maxima instead of significant changes in the absolute arrival time.

**Figure 3:**
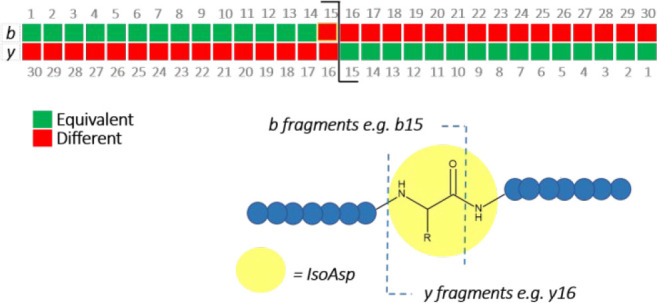
Schematic to illustrate the theoretical similarity of fragments in an isomerized peptide in comparison with a nonisomerized reference standard. Green squares are fragments that have an equivalent ATD to the RS19, and fragments with a red square are those that differ.

**Figure 4:**
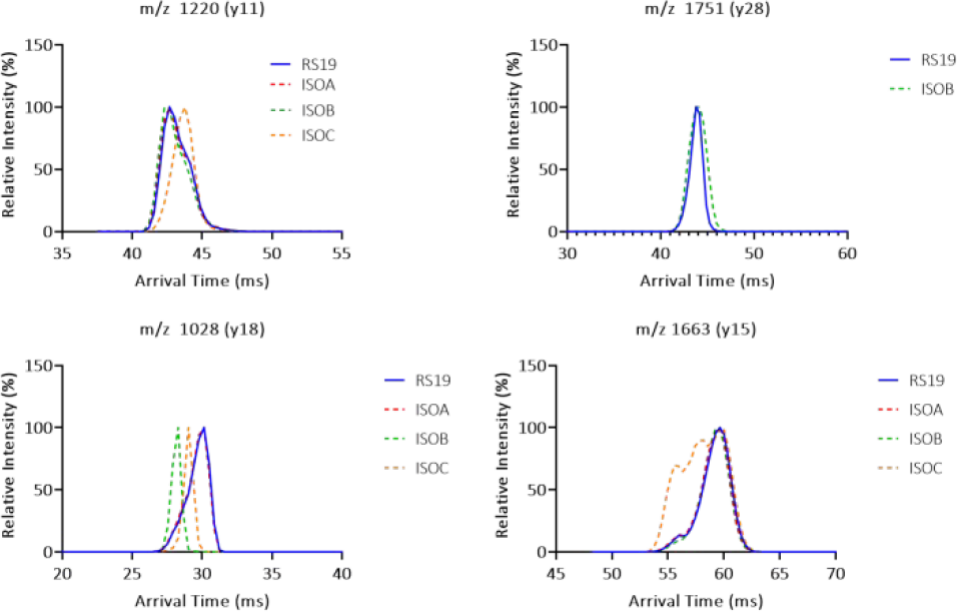
ATDs of fragment ions for RS19 and each isomer. For *y*11 it was expected and observed that the ATD for ISOA (red) and ISOB (green) would match RS19 (blue) but that ISOC (orange), containing the isoAsp, would have a different ATD. For fragment *y*18, it was expected and observed that only ISOA would match the ATD of RS19. For fragments *y*28 and *y*15 the change in the shape of the ATD was more pronounced than any change to the absolute arrival time.

Consequently, a pairwise comparison of the ATDs of isomer fragment ions with the ATDs of the nonisomerized RS19 fragment ions was undertaken, initially in a qualitative manner (by eye), to decide whether the ATD was “equivalent” or “different”. Figure 3 illustrates a theoretical fragment similarity pattern for an isomer at position 15, which in this case corresponds to ISOB. ATDs are predicted to be similar for *b*- and *y-*ions up to, but not including, fragments that contain the isoAsp, *b*1–14, or *y*1–15 (green, Figure 3). For isomerization at Asp9 (ISOA) and Asp21 (ISOC), the point at which “matching” fragments *switch* to “different” is also indicative of the fragmentation site proximity to the isomeric/structural change, and this is different for each isomer (Figure 4). A schematic of the instrument used in this study is shown in Figure S6 and depicts the SELECT SERIES Cyclic IMS with the cyclic T-wave device located upstream of the TOF mass analyzer. After quadrupole isolation (**1**) of the desired ions, CID in the trap generated a series of *b*/*y*-fragments (**2**). These fragments entered the cyclic device and were separated according to mobility (**3**) before reaching the high-field pusher of the TOF mass analyzer. Representative regions of fragmentation mass spectra are illustrated Figure S7. For ISOA, *y* fragments up to *y*21 were expected to be equivalent, for ISOB fragments up to *y*15 were expected to be equivalent, and for ISOC fragments up to *y*9 were expected to be equivalent.

Absolute arrival time differentiation (i.e., the ATD maximum) was not always observed, and in some cases fragments expected to be “similar” and “different” had approximately a 0.37 ms difference in arrival time. (Figure S8). This variability observed in absolute arrival times meant that this metric was not always sufficient to discern between “equivalent” and “different”. Figure 4 illustrates some mobilograms for fragments *y*11, *y*15, *y*18, and *y*28 of RS19 and the three isoAsp peptides detailing how isomer position changed the ATDs of the fragment ions. The fragment ions from *y*11 were all expected to be similar in conformation, and hence ATD, to RS19 except for that which contained an isoAsp. In this case, ISOC was the only fragment to be different which is consistent with the peptide being isoAsp21. The arrival time of ISOC *y*11 was 43.79 ms, and it was therefore proposed to be a larger, less compact ion than RS19 which had an arrival time of 42.68 ms. It is interesting to note that this is different to the results obtained for full-length precursor ions where RS19 had the least compact major conformation. For *y*18 both ISOC and ISOB were not equivalent to RS19; both had shorter arrival times and hence more compact structures. Interestingly, despite both containing an isoAsp, isoAsp21, and isoAsp15, respectively, the two *y*18 fragments of ISOB and ISOC were not equivalent to each other either, showing that the position of isomerization mattered even after CID fragmentation.

Fragment *y*28 (Figure 4) illustrated a typical challenge encountered when analyzing the data acquired in these experiments and, as mentioned before, some differences only manifested as changes to the shape of ATD and not the actual arrival time. An initial close inspection by eye of each fragment ion pair, across the entire series of each isomer, and extensive knowledge of the cIMS system, meant that the *y*28 fragment ions of RS19 and ISOB were initially qualitatively regarded as “different”. However, there was some ambiguity.

Hence, two mathematically simple approaches, subtraction and integration, were used to incorporate changes to the ATD shape into the analysis in order to distinguish isomer fragments further. Here, we propose that intensity normalization of isomer fragment ATDs (percentage intensity relative to maximum intensity of that fragment ion) followed by subtraction from the normalized RS19 mobilogram, then integration of the resulting curve to give an NSI value (normalization, subtraction integration, as described in the Experimental Section) is an appropriate metric. By normalizing the mobilograms to the highest peak intensity per fragment, the overall shape and arrival times were conserved enabling a quantitative comparison. This approach had the advantage of avoiding complications surrounding particular fragmentation efficiencies at different points in the sequence or slightly different peptide concentrations which might affect the overall number of ions produced per fragment.

**Figure 5:**
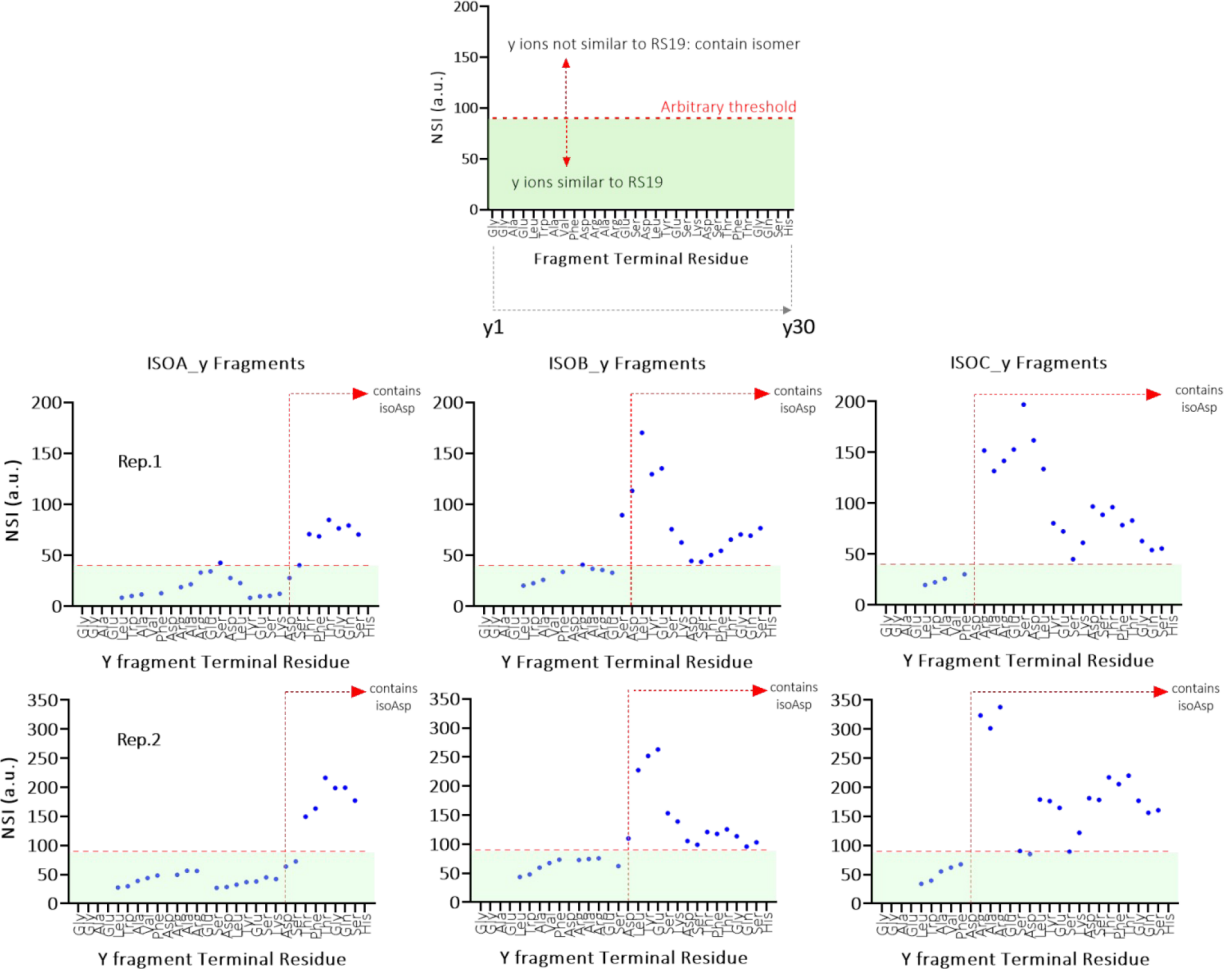
Pair-wise comparison of the running average of NSI per fragment plotted versus the residue at the terminus of each *y* fragment length. Example plot (top panel) illustrates an example with an arbitrary threshold dictating whether fragment ion ATDs were different or similar to the reference standard fragment ion. The six plots indicate that upon working from *y*1 – *y*30 (left to right), the first inflection in NSI value indicated correctly the fragment lengths which were conformationally different from the corresponding fragment in nonisomerized RS19. Hence, these were the fragments that contained the isoAsp. The experiment was run twice with two aliquots of the same samples, and data were acquired after only a single pass through the cyclic T-wave array. Red dashed vertical lines show the position of the isomerized Asp residue; therefore, fragments to the right of the line (longer *y* fragments) were expected, and observed, to have NSI values higher than this arbitrary threshold value (red horizontal line). The threshold value for replicate one is 40, and the value for replicate two is 90.

The NSI values were calculated (Tables S1–S3) for each fragment pair (isomer and RS19) and were added to a running average value (fragment *n*, *n* – 1, and *n* + 1), and this average value was then plotted for each fragment. In a plot of *y* fragment terminal residue vs NSI (Figure 5), an inflection point was apparent (sudden increase in NSI value) with one major transition observed per peptide indicating a significant conformational change had occurred in fragments containing that Asp residue only. Regarding the *b* fragment series, not as many of the fragments were observed but an increase in NSI was also seen for one replicate of ISOA (Figure S9). Thus, *y* ions were the focus of this study, given that they spanned almost the entire peptide sequence whereas *b* ions did not. The values around inflection points (Figure 5) were inspected to provide a guide toward establishing an arbitrary “threshold” value such that fragments with a running average NSI value above this were proposed to be significantly different. This analysis revealed that ISOA switched from “matching” to “different” at Asp9, ISOB at Asp15, and ISOC at Asp21. This was in agreement with the known identity of the peptides which had been synthesized with an isoAsp at each of these positions.

To assess day-to-day reproducibility of the approach, we performed the experiment twice at different times, months apart. Both experiments were performed as a single cIMS pass under similar conditions. Replicates 1 and 2 (Figure 5) displayed the expected NSI inflection points for all three isomers confirming the position of the isoAsp residue. However, some differences were observed between the replicates that are worth discussing. First, the threshold values are not quantitatively consistent between replicates 1 and 2, being 40 and 90, respectively. This can be accounted for by considering the high sensitivity of the NSI approach to small differences in the appearance of the ATD profiles. Identical ATDs would give NSI values of zero, but in reality the ion mobility data, even for equivalent ions, will have subtle differences that contribute to the NSI value. These differences might arise from arrival time drift between acquiring data for the RS19 peptide and the isomer peptide, possibly in response to environmental changes in the laboratory. Such a drift could be corrected either by CCS or “lock-drift” processing, but the format of the data makes this less facile than direct arrival time comparison. A second contributor to the threshold could be variations in ion statistics. If, for example, a particular ATD has fewer ion events to describe its shape, it may result in a larger NSI value when compared to an ATD for the same ion constituting a greater number of ion events. Such a situation might arise as a result of differences in acquisition time between RS19 and the isomer or differences in other instrumental conditions that may vary day-to-day. It is recommended, therefore, that each experiment should be analyzed as an individual data set and comparisons between replicates not be made, apart from the inflection point indicating the site of isomerization, which is consistent between replicates. It would not be appropriate to merge for example, some fragments from one acquisition with those from a separate acquisition of the same isomer.

Second, significant differences are observed between replicates for some NSI values for the same terminating residues. An example of this is exhibited most notably by ISOC *y*15 and *y*16 ions which in replicate 1 have running average NSI values of 197 and 162 and in replicate 2 have values of 90 and 85, respectively (Figure 5). This phenomenon is due to different product ion charge states in the different replicates being chosen for NSI processing. In replicate 1, only singly charged ions of *y*15 and *y*16 ions are compared between RS19 and ISOC, whereas in replicate 2 the doubly charged ions were chosen for these product ions (due to signal-to-noise considerations). Singly charged ions will likely have drastically different structures than doubly charged ions meaning the NSI values between any pair of ions will not be equivalent. It should be noted that we always selected the same ion to generate NSI values for each isomer *within* a replicate.

When evaluating trends across each sequence to identify the point of a conformational change, the use of NSI data without taking the running average along the fragment ion series, was considered. In this case, a threshold was difficult to determine because past the site of isomerization, the difference in NSI between isomer and RS19 decreased with increasing fragment length (Figure 5). This can be rationalized by considering that the optimal ion for differentiating fragments is when there are sufficient residues after the β-amino acid to translate into a conformational shift; but not too many residues such that the sensitivity to conformational changes is lost. This is a delicate balance to maintain as changes in conformation are relatively more significant in shorter fragments than longer ones.

Another significant point of note was regarding the lipidated fragments. Upon inspection of the two replicates for ISOA, a conformational change between isomer and reference was still detected despite the fact that the isomerized residue was directly adjacent to the lipidation site at Lys10. It was thought that fragments containing the lipid may not show any conformational differences arising from the isoAsp due to the high degree of flexibility of the lipid chain. However, this was not the case and for all three isomers, fragments that contained both isoAsp and the lipid were differentiated from their corresponding reference fragment. These results highlight the potential use of cIMS for peptide pharmaceuticals which are often lipidated analogues or conjugated to various polymers to optimize pharmacokinetics.^41,42^

Therefore, combining MS^2^ with the structure-based cIMS described here, enabled samples of four individual isomeric peptides to be identified from the ATDs of key fragment ions.

### LC-CID-MS Analysis of isoAsp Peptides

The characterization of therapeutic peptides and their impurities often relies on liquid chromatographic separation. However, retention time alone is not always sufficient for unambiguously assigning impurities such as isoAsp. While separation by LC is possible,^43,44^ subtle changes to mobile phases, e.g., pH, means the elution order from a mixed sample is not always diagnostic without first confirming peak identity using synthesized standards. There are, however, advantages to combining the above cIMS workflow with LC, not least of all is the automatability of LC as well as the potential for increased throughput for screening of many samples. Liquid chromatographic separation of peptides, prior to mobility separation and MS^2^ to generate an LC-CID-MS workflow, would allow more complex mixtures to be analyzed in a single experiment. Thus, determining if there were peptide impurities in a sample (LC), whether they were isoAsp isomers or chemical degradation products (MS) and also the location of the isomerized residue (CID-cIMS), if present.

**Figure 6:**
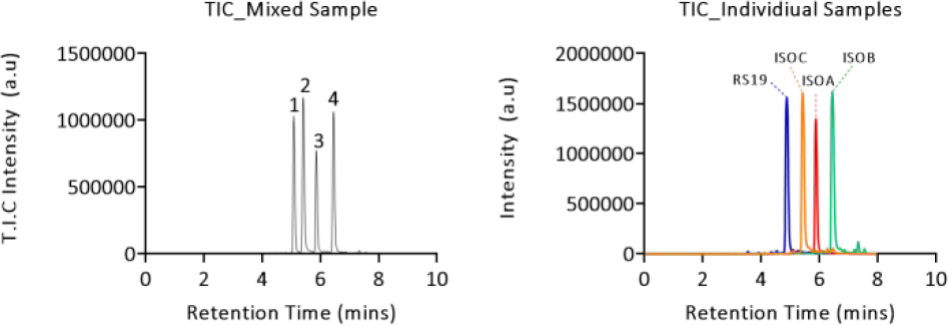
TIC chromatograms of peptide isomers in either a mixed sample (left) which would not be unambiguously identified by MS^2^ alone and the TIC chromatogram of samples individually submitted to LC (right) which allowed correlation of cIMS fragment ion assignments to known retention times for each isomer.

**Figure 7:**
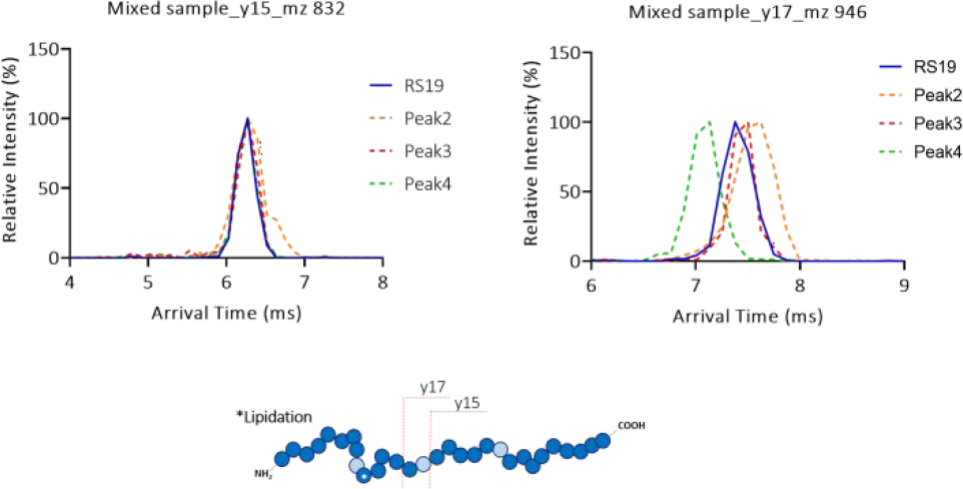
ATDs generated from fragment ions of each peak in the LC chromatogram corresponding to each peptide fragment *y*17 (left) or *y*15 (right). After elution from the columns the samples were subjected to CID fragmentation (45 V) followed by a single pass around the cIMS T-wave array.

The same NSI analysis was conducted for samples of each peptide submitted to LC prior to CID-cIMS individually as well as a sample of each peptide mixed (25% v/v) (Figure 6). For one of the UPLC peaks in the mixed sample (ISOA) there was the characteristic spike in the running average NSI cIMS data for *y* fragments, as one would expect for isomerization at Asp9 (Figure S10). Thus, ISOA was easily identified from the heterogeneous sample. The retention time of RS19 was known, as would be the case in a typical drug development program and by extension the remaining choice of which peak was which between ISOB and ISOC (Figure 7). Inspection of the *y* fragment data for the remaining two peaks was ambiguous, as not all of the key fragments were observed. With pure samples of each isomer available, comparison of retention times was used to decipher peak 4 from peak 2, although, inspection of the running average NSI values for peak 4 did give an inflection point where it would be expected for isomerization at Asp15 residue. Despite this, after LC separation it was not possible to unambiguously assign ISOC without observing fragments in the range of *y*6–14.

However, differentiation was indeed possible by analysis of specific fragment ion ATDs alone, rather than the running average method described above. Inspection of fragment ion peaks for *y*15 and *y*17 allowed speculation as to which peak belonged to which isomer as a result of the sensitivity of the cIMS instrument after a single pass. If the peptide was isomerized at Asp9, then both *y*15 and *y*17 would match; this corresponds to the red trace in Figure 7. If the isomerized peptide was isoAsp21, then it was expected that both fragments would be different. and this was observed; see the orange trace (peak 2). However, the green trace for peak 4 (Figure 7) was seen to change from matching at the *y*15 fragment length but was considerably different for the *y*17 fragment. This would be consistent with what was expected for isomerization at Asp15. Therefore, while the fragmentation post LC requires optimization to observe the entire *y* fragment series, there is significant promise for using LC-CID-cIMS for the identification of unknown isoAsp peptides from a sample containing a mixture of all isomers and the nonisomerized reference standard for the therapeutic peptide of interest.

## Conclusion

Despite their propensity for aggregation, peptides play an increasingly important role as therapeutics sitting between the pharmacokinetic stereotypes of small molecules and large biomolecules. As such, analytical platforms able to support these modalities are becoming increasingly important. IMS technology has advanced to such an extent that even conformational subtleties, like those imparted by amino acid isomerization, can be measured. Thus, there is an opportunity to now use such techniques to develop accurate and powerful methods to detect and characterize even small differences in therapeutic peptides. As part of this process, the search for simple yet accurate methods for comparison of ATD ion mobility data from different peptides is of paramount importance. In this study, we chose a mathematically facile approach to transition from qualitative assessment of ATD differences to a more objective and quantitative framework. Here, we have demonstrated that the high resolution and sensitivity of cIMS, in conjunction with a simple normalization-subtraction-integration (NSI) approach to data processing, has the ability to differentiate *and* identify the site of isomerization in a series of three synthetic isobaric peptides based on an important therapeutic scaffold. This approach has significant benefit over other conventional solution ensemble techniques which cannot always identify these impurities with sufficient speed or resolution. Movement toward higher resolution techniques like cIMS will vastly improve our understanding of the potential impact of degradants/impurities on the physical stability of a peptide and factors affecting its shelf life. This capability for conformation-based differentiation using cIMS together with mass spectrometry is a major step forward for the characterization of chemically similar and isobaric but structurally different biopharmaceutical impurities, which is crucial for ensuring safety and quality control of peptide therapeutics.

## Supporting Information Available

Additional ATDs, mass spectra, and processed NSI data (PDF)

## Notes

Processed data for figures and tables in the manuscript is available on request by emailing viv.lindo@astrazeneca.com
