# Supplementary material for: Toward Rapid Aspartic Acid Isomer Localization in Therapeutic Peptides Using Cyclic Ion Mobility Mass Spectrometry
Source: J Am Soc Mass Spectrom. 2022 May 24;33(7):1204–12. doi: 10.1021/jasms.2c00053 (PMC9264384; doi:10.1021/jasms.2c00053)
Supplement: Supplementary file 1 — js2c00053_si_001.pdf [file js2c00053_si_001.pdf]

Supporting Information

Towards rapid aspartic acid isomer localization in therapeutic peptides using cyclic ion mobility mass spectrometry

Katherine Gibson [‡ ±], Dale A. Cooper-Shepherd [Ø], Edward Pallister[±], Sophie E. Inman[±], Sophie E. Jackson[‡*] and Viv Lindo[± *]

‡ Yusuf Hamied Department of Chemistry, University of Cambridge, Cambridge, CB2 1EW, U.K.

± Analytical Sciences, BioPharmaceuticals Development, R&D, AstraZeneca, Cambridge, CB21 6GH, U.K.

Ø Waters Corporation, Wilmslow, SK9 4AX, U.K

*Corresponding Authors: S.E. Jackson (sej13@cam.ac.uk and V. Lindo (viv.lindo@astrazeneca.com)

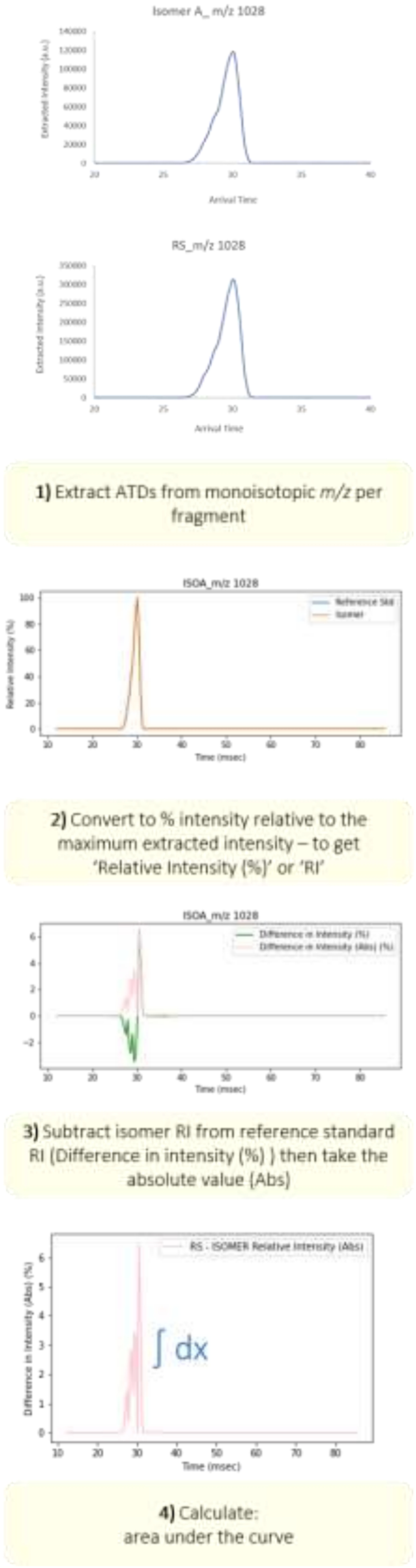

Figure S1: Workflow for the normalisation and processing of fragment ATD data before calculating the NSI and running average NSI.

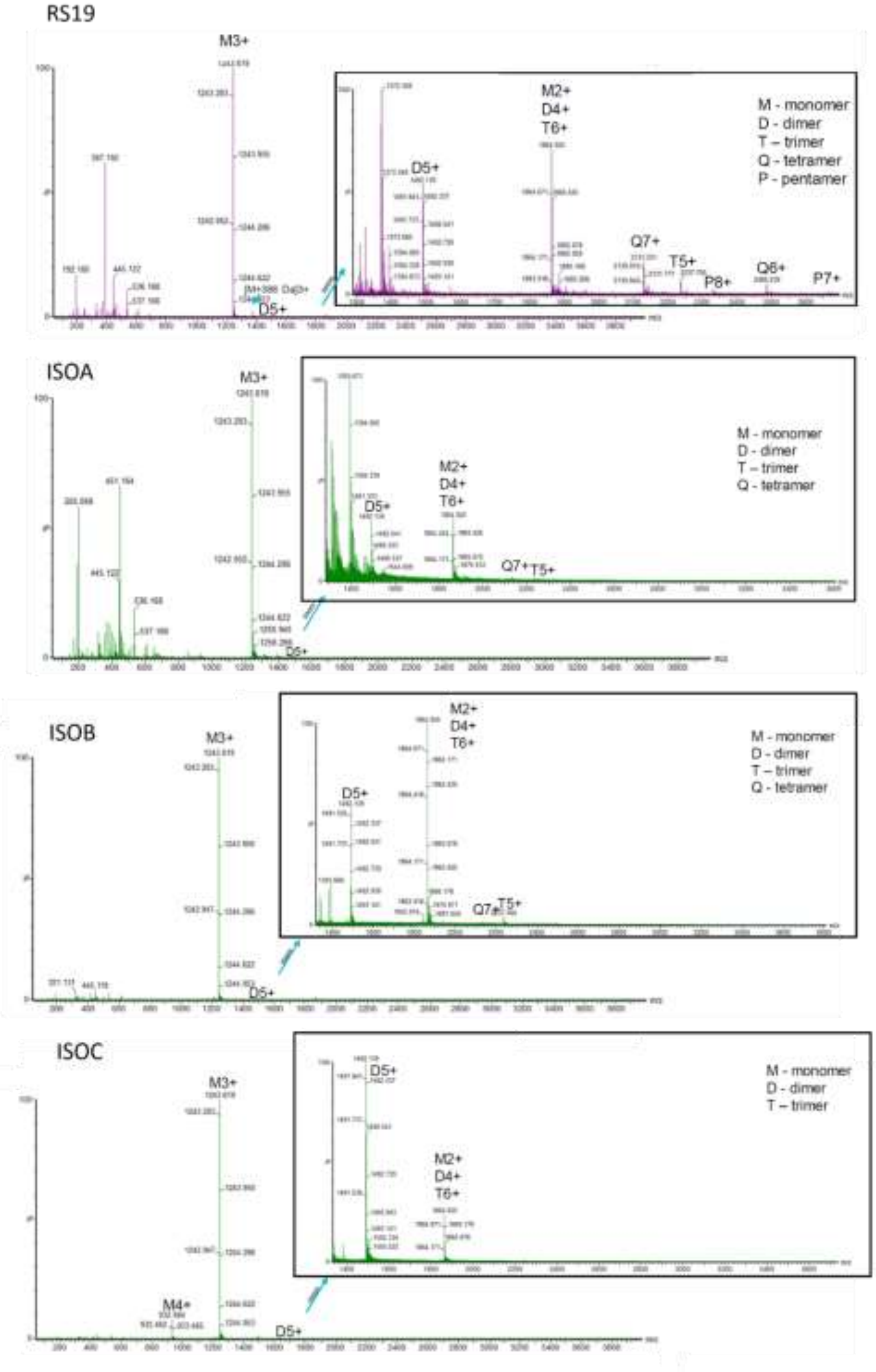

Figure S2: Native mass spectra of the three isomers ISOA (5 mM), ISOB (9 mM) ISOC (9 mM) and RS19 (8 mM) sprayed using nESI and the Waters Select Cyclic series. All isomers have a preference for the +3 charge state (m/z 1242.9) but only RS19 showed a significant proportion of oligomers. ISOC was the only isomer to ionize well in the +4 protonated charge state (m/z 932.4). M= monomer, D= dimer, T= trimer, Q= tetramer,

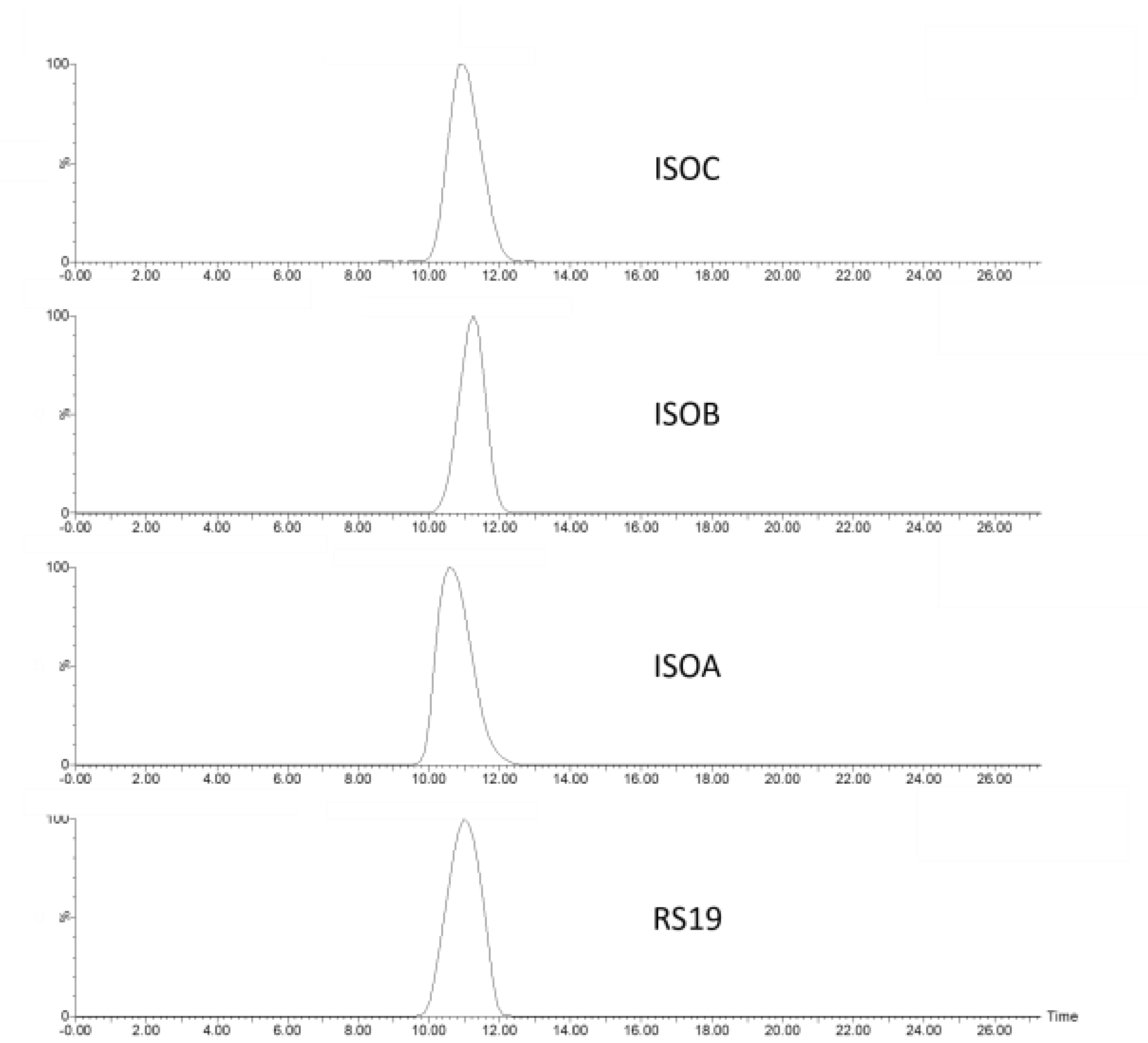

Figure S3: ATD data of aliquots from the same preparation as the cIMS data, except acquired using the linear Synapt XS instrument. Samples were thawed once, as with cIMS, and run using the following parameters: source temperature 60 °C, sampling cone 35 V, IMS wave velocity 850 m/s and wave height of 34 V. Samples were infused using the TriVersa Nanomate (Advion) and ESI chip with nominal 5.5 µm nozzle emitters using a spray voltage of 1.7 kV. All the x-axes are in ms.

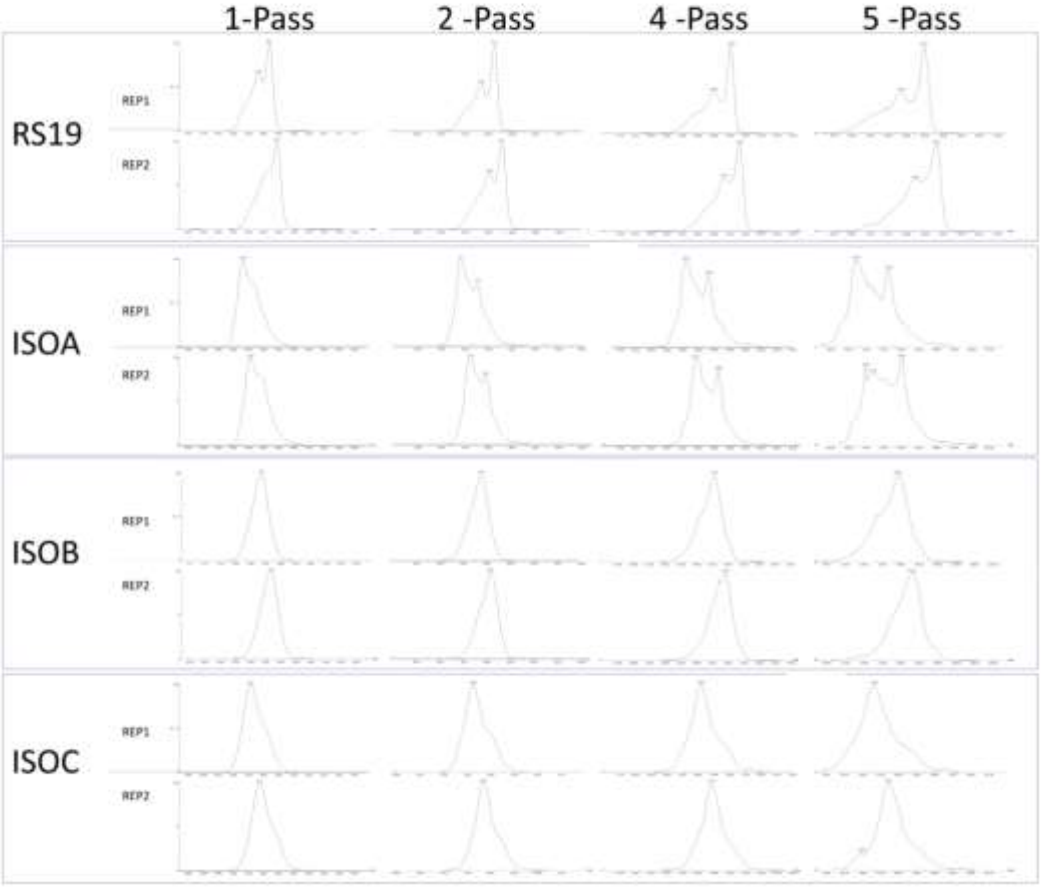

Figure S4: Reproducibility of the ATDs generated for 1,2,4 and 5 passes around the cyclic IMS. A single dataset was collected on two separate days and the ATDs compared. The data are subject to slight ATD drift but the overall shape of each ATD remains similar and the four isomers distinguishable after 4 passes. The x-axes are all in ms.

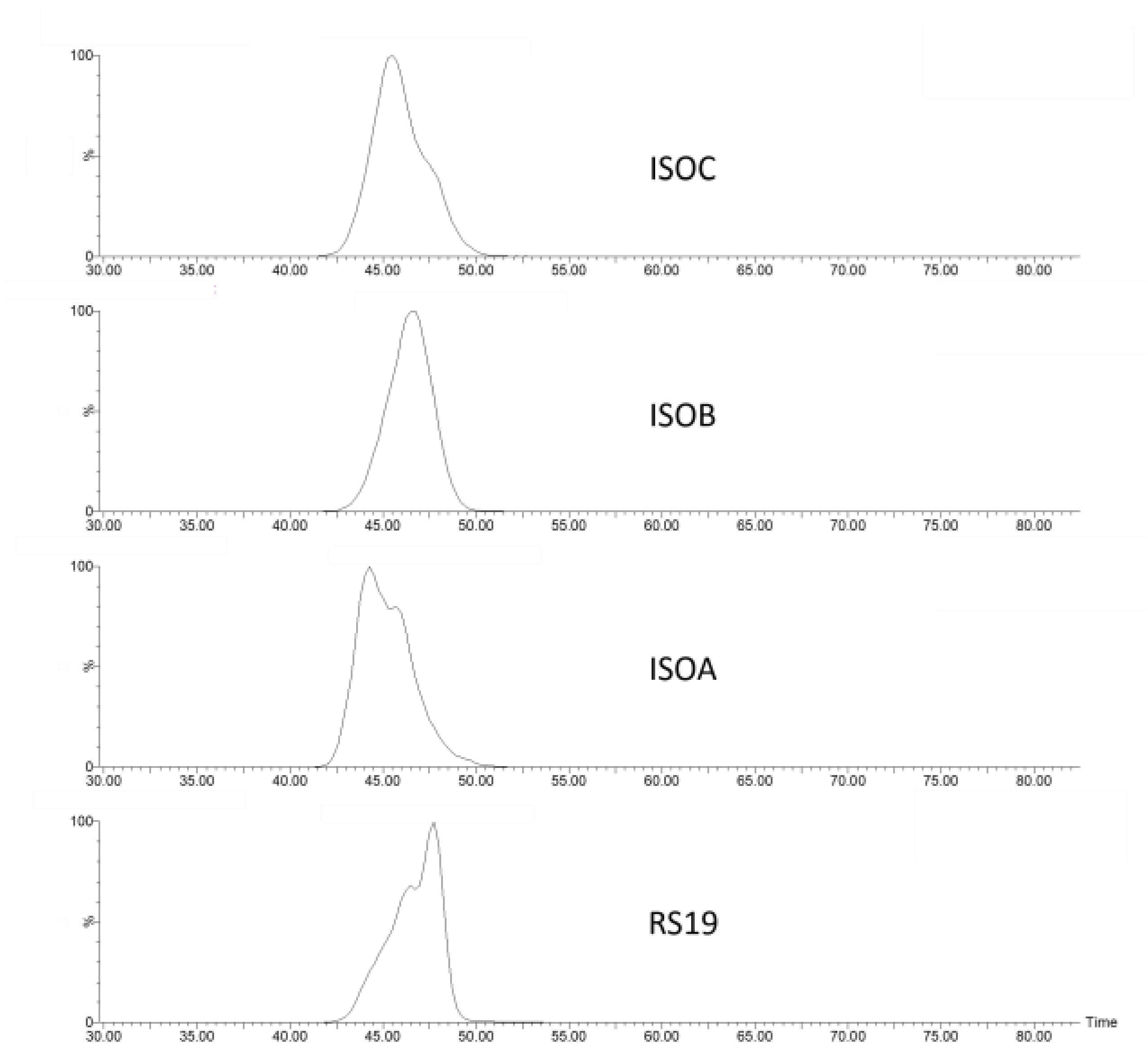

Figure S5: Single pass cIMS data for each isomer ranging in concentration from 5 – 9 mM for comparison to the multipass data shown in Fig. 2. The x- axis is in milliseconds and spans approximately 52 msec, similar to the x-axis for the 4-pass data in Figure 2 c.

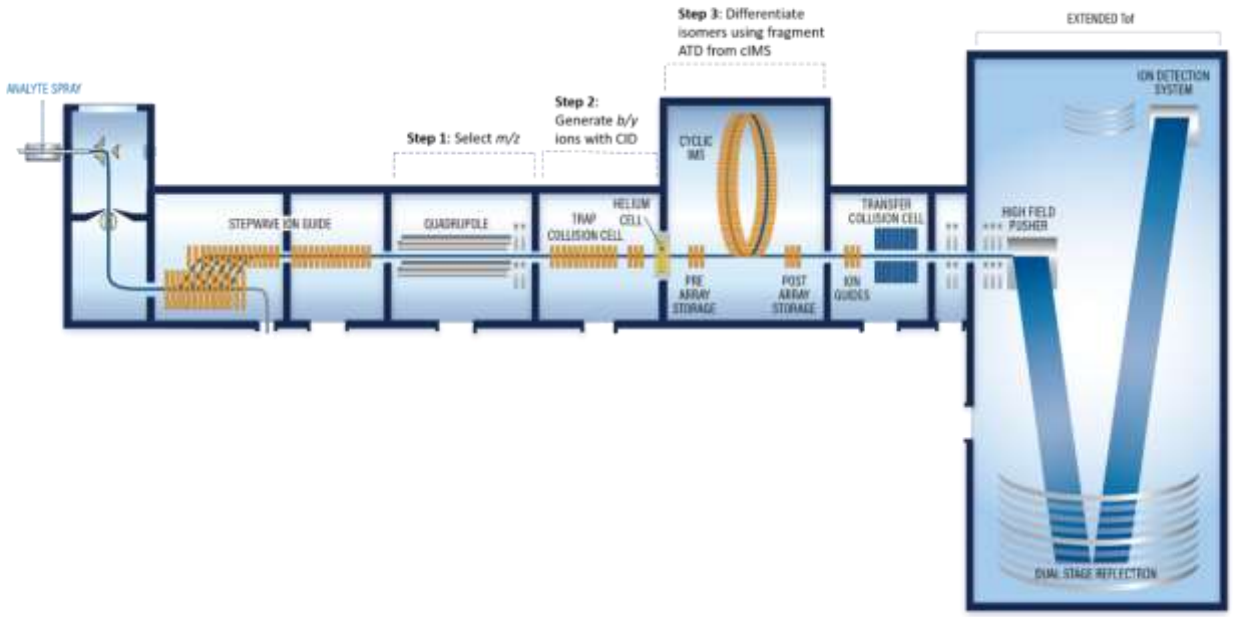

Figure S6: Schematic diagram of the SELECT SERIES cyclic IMS with the Cyclic T-wave device located upstream of the TOF mass analyser. After quadrupole isolation **(1),** of the desired ions, collision-induced dissociation (CID) in the trap region generated a series of b/y-fragments **(2).** These fragments entered the cIMS module of the instrument and were separated according to mobility **(3)**, before reaching the high-field pusher of the TOF mass analyser.

The four base spectra spectra zoomed by 50%

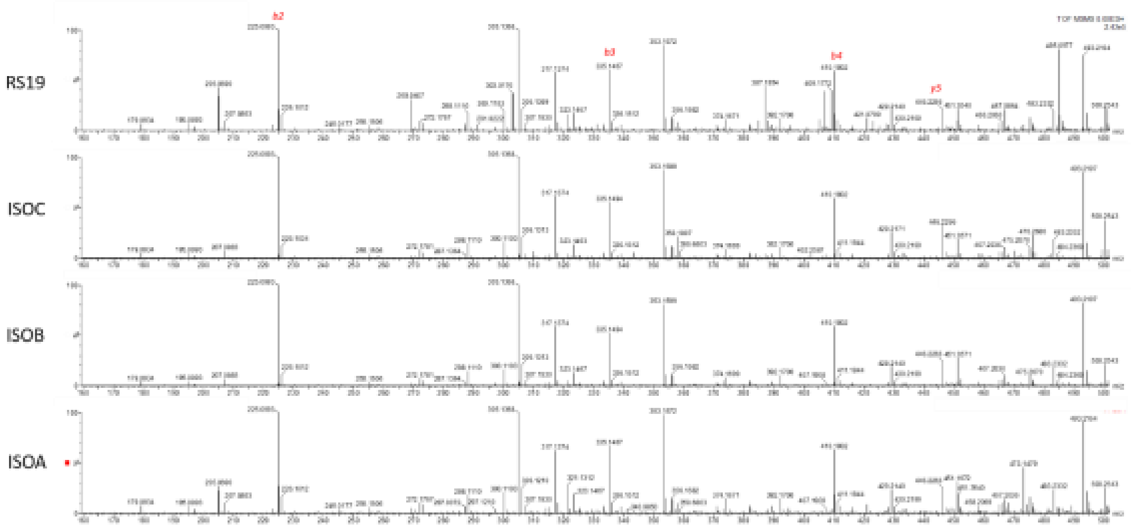

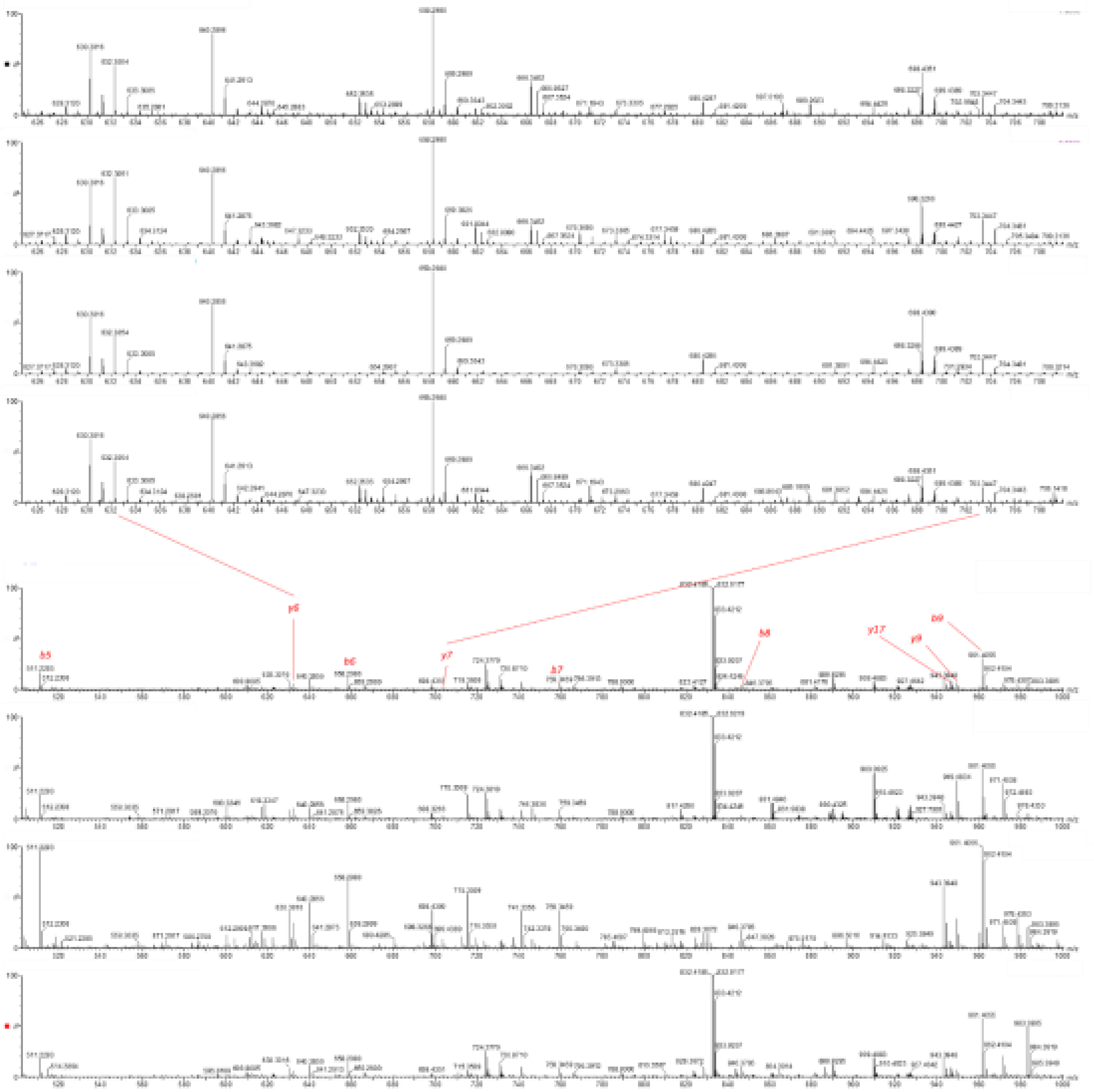
b5
y6
b6
y7
b7
b8
y17
y9
b9

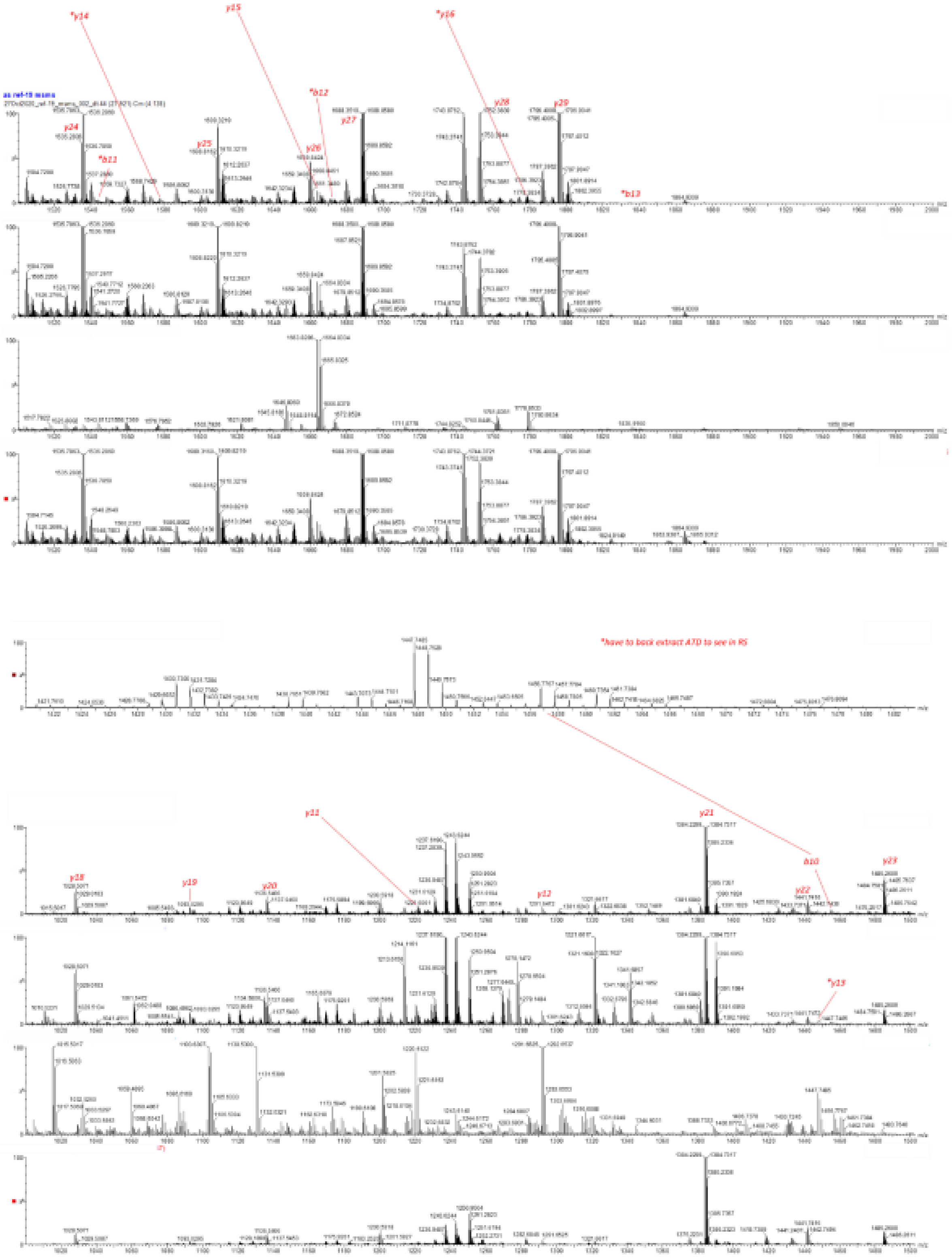

Figure S7: Annotated fragmentation spectra of each isomer after direct infusion of individual samples. Each spectra has been zoomed by approximately 50% when appropriate to facilitate annotation. The x-axes show the m/z values.

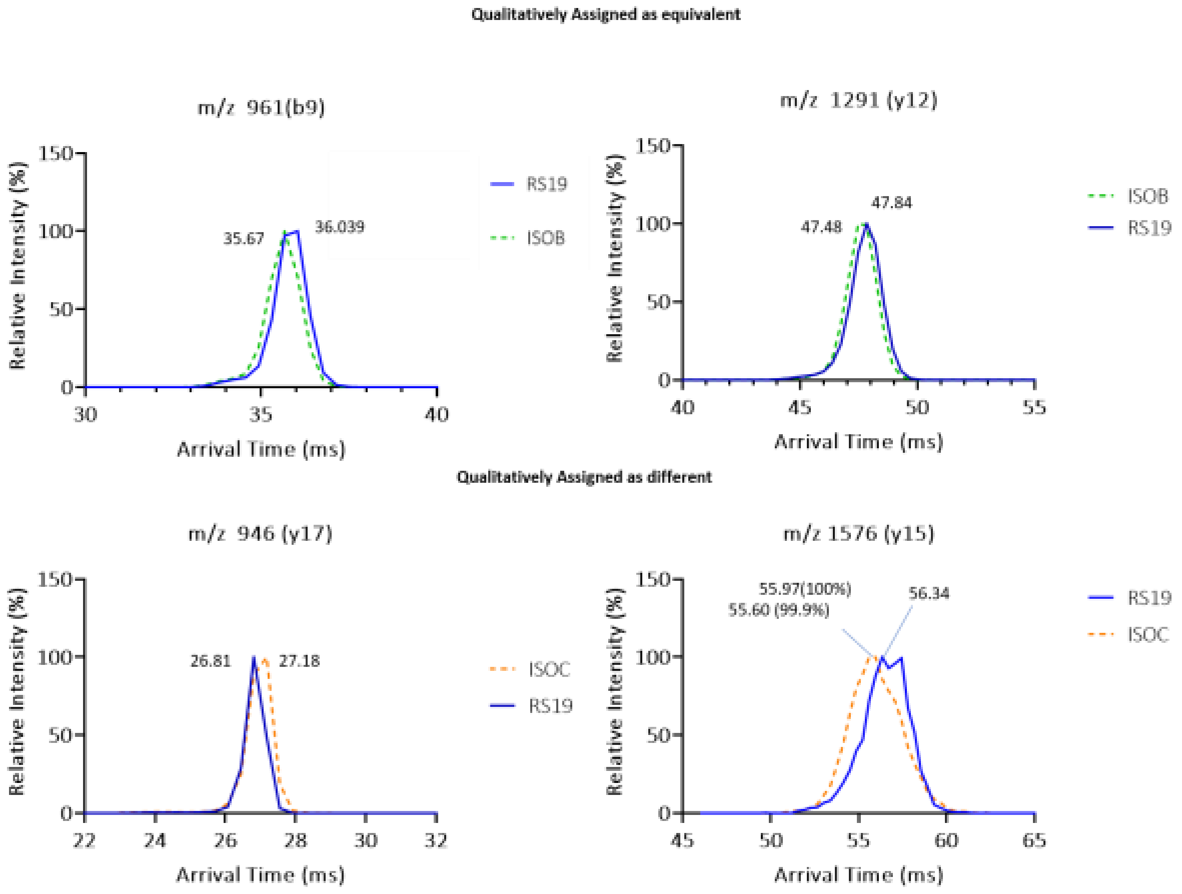

Figure S8: Top – fragment ions which were assigned as equivalent by qualitative assessment of the data. Bottom -fragment ions qualitatively assessed as different. Qualitative assessment of the ATDs was able to account for peak shape and asymmetry and width despite very similar arrival time shifts for equivalent and different fragments. For m/z 1576 and ISOC, the peak spans arrival times from 55.60 to 55.97 ms, the latter being within 0.37 ms of the RS19 peak, which is similar to the fragments shown in the top panel which were assigned as equivalent. MassLynx reports only the first arrival time, 55.60 ms, as the peak maximum. However, it is clear from m/z 1576 and ISOC / RS19 data that the ATDs are different. Thus, when the peak is rather flat, the selection of the maximum by MassLynx is somewhat variable.

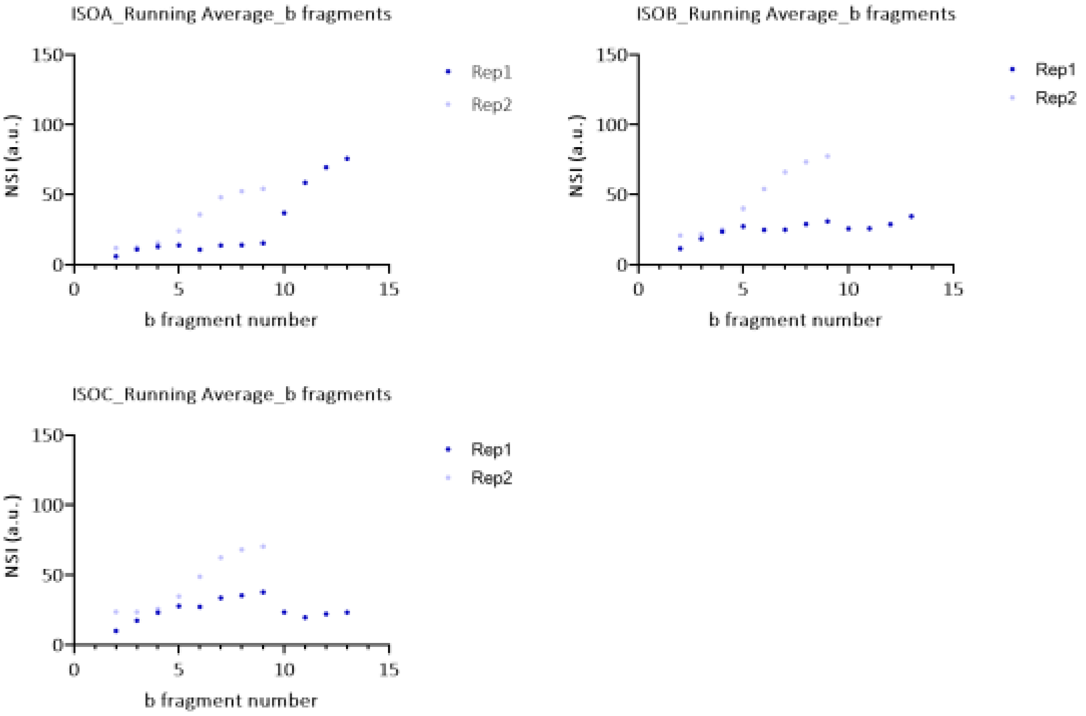

Figure S9: b fragment ion ATD for each isomer and each replicate. These ions were not as informative as the y fragment ion series, and less than half of the sequence was identified hence they were not correlated to a change in conformation therefore could not be used to identify the position of the IsoAsp.

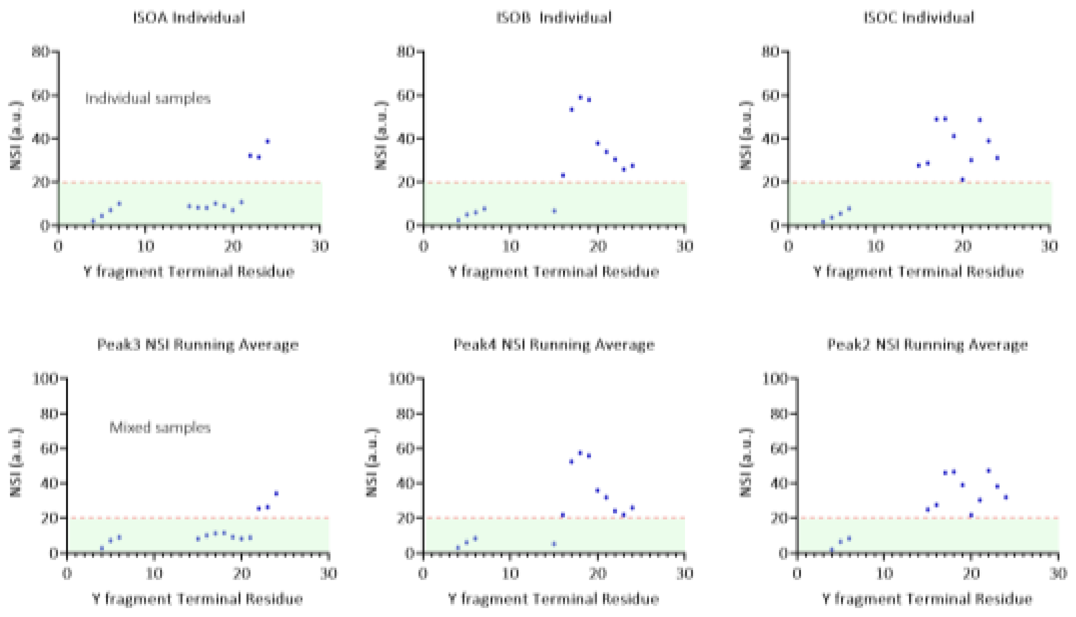

Figure S10: Comparison of the running average of NSI values for samples individually submitted to a LC-cIMS-MS (top) and those submitted from a mixture of all four peptides in one sample (bottom). From the UV retention time of RS19 individually submitted, it was known which peak was RS19 in the mixed sample and hence fragment ions from each peak were compared to that of RS19 = peak 1 in this notation. Peaks were numbered 1-4 from the fastest retention time to the slowest.

Table 1: Running average NSI values for isomer A (ISO9). NSI values (arbitrary units) for each y fragment observed in the two replicates.

						ISOA						
Rep1				NSI	Running Average		Rep2				NSI	Running Average
	Y Fragment Terminal Amino Acid	Y Fragment	m/z (obs. RS19)					Y Fragment Terminal Amino Acid	Y Fragment	m/z (obs. RS19)		
	30	His						30	His			
	29	Ser	1795.4005	59.19	70.435			29	Ser	1795.3977	144.76	177.265
	28	Gln	1751.8807	81.68	79.19333			28	Gln	1751.8824	209.77	199.4733
	27	Gly	1687.8521	96.71	76.40333			27	Gly	1687.8542	243.89	198.797
	26	Thr	1659.3408	50.82	84.79333			26	Thr	1659.3401	142.73	216.34
	25	Phe	1608.8162	106.85	68.63667			25	Phe	1608.8207	262.4	163.53
	24	Thr	1535.2806	48.24	70.71333			24	Thr	1535.2811	85.47	149.3433
	23	Ser	1484.7581	57.05	40.08			23	Ser	1484.7581	100.16	72.37667
	22	Asp	1441.2407	14.95	27.83			22	Asp	1441.2396	31.5	63.76
	21	Lys	1383.728	11.49	12.31			21	Lys	1383.7274	59.62	42.02
	20	Ser	1136.0426	10.49	10.21			20	Ser	1136.0419	34.94	45.08333
	19	Glu	1092.5255	8.65	9.76			19	Glu	1092.5292	40.69	37.97333
	18	Tyr	1028.004	10.14	8.206667			18	Tyr	1028.0049	38.29	36.79
	17	Leu	946.4727	5.83	22.68333			17	Leu	946.4728	31.39	32.50667
	16	Asp	1778.8533	52.08	27.72			16	Asp	889.931	27.84	28.45333
	15	Ser	1663.8286	25.25	42.58			15	Ser	832.4174	26.13	26.985
	14	Glu	1576.7893	50.41	34.22			14	Glu			
	13	Arg	1447.7485	27	33.09			13	Arg	1447.7523	70.43	56.23
	12	Ala	1291.6472	21.86	21.54333			12	Ala	1291.6508	42.03	56.36333
	11	Arg	1220.6122	15.77	18.815			11	Arg	1220.6125	56.63	49.33
	10	Asp						10	Asp			
	9	Phe	949.4834	12.67	12.67			9	Phe	949.4832	54.31	48.22
	8	Val						8	Val	802.4157	42.13	43.85
	7	Ala	703.3447	13.49	11.605			7	Ala	703.3449	35.11	39
	6	Trp	632.3054	9.72	10.09			6	Trp	632.3054	39.76	29.99333
	5	Leu	446.2266	7.06	8.39			5	Leu	446.2288	15.11	27.435
	4	Glu						4	Glu			
	3	Ala						3	Ala			
	2	Gly						2	Gly			
	1	Gly						1	Gly			

Table 2: Running average NSI values for isomer B (ISO15) NSI values (arbitrary units) for each y fragment observed in the two replicates.

						ISOB						
Rep1				NSI	Running Average		Rep2				NSI	Running Average
	Y Fragment Terminal Amino Acid	Y Fragment	m/z (obs. RS19)					Y Fragment Terminal Amino Acid	Y Fragment	m/z (obs. RS19)		
	30	His						30	His			
	29	Ser	1795.4005	80.08	76.585			29	Ser	1795.3977	74.14	103.06
	28	Gln	1751.8807	73.09	69.25			28	Gln	1751.8824	131.98	95.81333
	27	Gly	1687.8521	54.58	70.47			27	Gly	1687.8542	81.32	113.71
	26	Thr	1659.3408	83.74	65.45667			26	Thr	1659.3401	127.83	125.6067
	25	Phe	1608.8162	58.05	54.44333			25	Phe	1608.8207	167.67	117.7267
	24	Thr	1535.2806	21.54	50.28667			24	Thr	1535.2811	57.68	120.81
	23	Ser	1484.7581	71.27	43.59333			23	Ser	1484.7581	137.08	99.00667
	22	Asp	1441.2407	37.97	44.35			22	Asp	1441.2396	102.26	105.3367
	21	Lys	1383.728	23.81	62.53667			21	Lys	1383.7274	76.67	139.0667
	20	Ser	1136.0426	125.83	75.57			20	Ser	1136.0419	238.27	153.32
	19	Glu	1092.5255	77.07	135.2967			19	Glu	1092.5292	145.02	262.9633
	18	Tyr	1028.004	202.99	129.6333			18	Tyr	1028.0049	405.6	251.8867
	17	Leu	946.4727	108.84	170.22			17	Leu	946.4728	205.04	227.22
	16	Asp	1778.8533	198.83	113.1867			16	Asp	889.931	71.02	109.7633
	15	Ser	1663.8286	31.89	89.41333			15	Ser	832.4174	53.23	62.125
	14	Glu	1576.7893	37.52	32.87			14	Glu			
	13	Arg	1447.7485	29.2	35.52667			13	Arg	1447.7523	77.72	75.615
	12	Ala	1291.6472	39.86	36.80667			12	Ala	1291.6508	73.51	74.39333
	11	Arg	1220.6122	41.36	40.61			11	Arg	1220.6125	71.95	72.73
	10	Asp						10	Asp			
	9	Phe	949.4834	33.74	33.74			9	Phe	949.4832	87.95	73.28
	8	Val						8	Val	802.4157	58.61	67.62667
	7	Ala	703.3447	27.04	25.955			7	Ala	703.3449	56.32	59.68333
	6	Trp	632.3054	24.87	22.54			6	Trp	632.3054	64.12	47.84667
	5	Leu	446.2266	15.71	20.29			5	Leu	446.2288	23.1	43.61
	4	Glu						4	Glu			
	3	Ala						3	Ala			
	2	Gly						2	Gly			
	1	Gly						1	Gly			

Table 3: Running average NSI values for isomer C (ISO21) NSI values (arbitrary units) for each y fragment observed in the two replicates.

ISOC												
Rep1				NSI	Running Average		Rep2				NSI	Running Average
	Y Fragment Terminal Amino Acid	Y Fragment	m/z (obs. R519)					Y Fragment Terminal Amino Acid	Y Fragment	m/z (obs. R519)		
	30	His						30	His			
	29	Ser	1795.4005	36.95	55.52			29	Ser	1795.3977	120.84	160.7
	28	Gln	1751.8807	74.09	54.10333			28	Gln	1751.8824	200.56	156.0767
	27	Gly	1687.8521	51.27	62.93			27	Gly	1687.8542	146.83	176.9267
	26	Thr	1659.3408	63.43	83.08			26	Thr	1659.3401	183.39	219.9333
	25	Phe	1608.8162	134.54	78.65667			25	Phe	1608.8207	329.58	205.58
	24	Thr	1535.2806	38	96.18667			24	Thr	1535.2811	103.77	217.07
	23	Ser	1484.7581	116.02	88.77			23	Ser	1484.7581	217.86	178.2433
	22	Asp	1441.2407	112.29	96.85			22	Asp	1441.2396	213.1	181.1333
	21	Lys	1383.728	62.24	61.21333			21	Lys	1383.7274	112.44	121.6933
	20	Ser	1136.0426	9.11	45.07667			20	Ser	1136.0419	39.54	89
	19	Glu	1092.5255	63.88	72.42333			19	Glu	1092.5292	115.02	164.73
	18	Tyr	1028.004	144.28	80.34333			18	Tyr	1028.0049	339.63	176.44
	17	Leu	946.4727	32.87	133.7567			17	Leu	946.4728	74.67	178.9667
	16	Asp	1778.8533	224.12	161.95			16	Asp	889.931	122.6	85.10333
	15	Ser	1663.8286	228.86	197.1433			15	Ser	832.4174	58.04	90.32
	14	Glu	1576.7893	138.45	152.8867			14	Glu			
	13	Arg	1447.7485	91.35	141.8067			13	Arg	1447.7523	257.45	337.84
	12	Ala	1291.6472	195.62	131.7267			12	Ala	1291.6508	418.23	301.5267
	11	Arg	1220.6122	108.21	151.915			11	Arg	1220.6125	228.9	323.565
	10	Asp						10	Asp			
	9	Phe	949.4834	30.18	30.18			9	Phe	949.4832	66.84	67.575
	8	Val						8	Val	802.4157	68.31	61.98333
	7	Ala	703.3447	27.85	25.995			7	Ala	703.3449	50.8	55.39
	6	Trp	632.3054	24.14	22.44333			6	Trp	632.3054	47.06	39.6
	5	Leu	446.2266	15.34	19.74			5	Leu	446.2288	20.94	34
	4	Glu						4	Glu			
	3	Ala						3	Ala			
	2	Gly						2	Gly			
	1	Gly						1	Gly			
